# Supervised Therapeutic Leave and Relapse Outcomes Following Discharge from a Mandated Addiction Rehabilitation Programme in Qatar: A Retrospective Time-to-Event Study

**DOI:** 10.1192/bjo.2026.11133

**Published:** 2026-06-30

**Authors:** Faycal Walid Ikhlef, Nirvana Swamy Kudlur Chandrappa, Mugtaba Osman, Suhair Mohammed Yousuf, Ahmad Maaen Alater

**Affiliations:** Mental Health Services HMC, Doha, Qatar

## Abstract

**Aims:**

To assess the association between access to supervised therapeutic leave during mandated inpatient substance use disorder rehabilitation and post-discharge relapse outcomes in a national cohort in Qatar. Specifically, the study examines time to relapse following discharge and explores early recovery stability during the transition from inpatient care to the community.

**Methods:**

A retrospective cohort study was conducted using medical records of 72 patientsdischarged from a mandated residential rehabilitation service in 2023. Exposure was defined as receipt of any supervised therapeutic leave during admission. Relapse was defined as a positive urine drug screen during the 26-week follow-up period. Time-to-relapse was analysed using Kaplan–Meier methods, with between-group comparisons conducted using log-rank testing. Multivariable Cox proportional hazards modelling adjusted for demographic and clinical covariates, including comorbid personality disorder and forensic history.

**Results:**

Twenty-eight patients (38.9%) received supervised therapeutic leave, while 44 (61.1%) did not. Relapse occurred in 40.3% of the cohort, with a median time-to-relapse of 28 days. Patients granted therapeutic leave demonstrated significantly longer relapse-free survival than those without leave (log-rank p=0.034). During follow-up, relapse was observed in 25% of patients who received therapeutic leave compared with 50% among those without leave. After adjustment for covariates, supervised therapeutic leave was independently associated with a marked reduction in relapse hazard (hazard ratio 0.27; p=0.0088). Greater frequency of leave exposure was associated with lower relapse risk, suggesting a graded protective effect.

**Conclusion:**

As one of the first evaluations of supervised therapeutic leave within a compulsory addiction treatment model in the region, these findings indicate that structured community leave may meaningfully enhance early recovery stability and delay relapse following discharge. Integrating carefully governed therapeutic leave into mandated treatment pathways may strengthen reintegration while maintaining clinical and public safety safeguards during the critical post-discharge period.

